# The role of statisticians in the response to COVID-19 in Israel: a holistic point of view

**DOI:** 10.1186/s13584-022-00531-y

**Published:** 2022-04-20

**Authors:** Itai Dattner, Reuven Gal, Yair Goldberg, Inbal Goldshtein, Amit Huppert, Ron S. Kenett, Orly Manor, Danny Pfeffermann, Edna Schechtman, Clelia di Serio, David M. Steinberg

**Affiliations:** 1 University of Haifa, Haifa, Israel; 2 Samuel Neaman Institute, Technion, Haifa, Israel; 3 Technion, Haifa, Israel; 4 MaccabiTech, Tel Aviv, Israel; 5 Gertner Institute, Ramat Gan, Israel; 6 KPA Group, Ra’anana, Israel; 7 University of Turin, Turin, Italy; 8 Hebrew University, Jerusalem, Israel; 9 Israel Central Bureau of Statistics, Jerusalem, Israel; 10 University of Southampton, Southampton, UK; 11 Ben Gurion University of the Negev, Be’er Sheva, Israel; 12 Università Vita-Salute San Raffaele, Milan, Italy; 13 Tel Aviv University, Tel Aviv, Israel

**Keywords:** Statistics, Data analysis, Data collection, Data quality, Modeling, Pandemic, Data driven policy

## Abstract

The COVID-19 pandemic cast a dramatic spotlight on the use of data as a fundamental component of good decision-making. Evaluating and comparing alternative policies required information on concurrent infection rates and insightful analysis to project them into the future. Statisticians in Israel were involved in these processes early in the pandemic in some silos as an ad-hoc unorganized effort. Informal discussions within the statistical community culminated in a roundtable, organized by three past presidents of the Israel Statistical Association, and hosted by the Samuel Neaman Institute in April 2021. The meeting was designed to provide a forum for exchange of views on the profession’s role during the COVID-19 pandemic, and more generally, on its influence in promoting evidence-based public policy. This paper builds on the insights and discussions that emerged during the roundtable meeting and presents a general framework, with recommendations, for involving statisticians and statistics in decision-making.

## Introduction

The COVID-19 pandemic posed significant challenges to the health, economic and social systems around the globe. Faced with a crisis at a scale not experienced in the last century, policy makers were required to make difficult decisions on a daily basis. Ideally, such policy decisions should be based on properly analyzed data and subject knowledge. Specifically, decision makers should seek actionable information coming from robust and rigorous analysis of accurate and relevant data. In reality, specific policy choices varied widely, from the airtight seal in New Zealand to the lockdowns in Europe and the laisse faire attitude of Brazil. Countries veered from one policy to another. For example, Britain adopted tight lockdowns only after a limited social distancing campaign was accompanied by heavy infection and burden of disease. In Israel, infections waned following a tight lockdown, but returned to much higher levels several months after most restrictions were removed.

As a pandemic unfolds, new dilemmas arise. Depending on the state of emergency, tools being used and depth of analysis will vary. But in any case, a volatile reality as manifested in crisis times requires a well-founded and robust approach for data-driven decision making. The ability to rapidly obtain high quality data and to analyze it, in a timely manner, cannot be taken for granted. In fact, in many countries there is uncertainty about the most basic issue—how many people died as a result of COVID-19 infection. In Russia, for example, “data-based” estimates have suggested that the true death count may be twice as high as the official count [1], and in India the assessed gap is even larger [2].

**Fig. 1:**
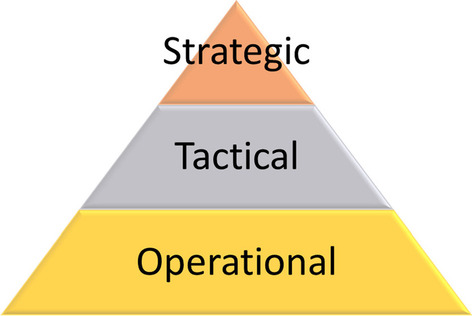
Decision making domains requiring complementary statistical skills

Successfully addressing challenges, like those mentioned above, requires among other things, *statistical thinking* leading to sound statistical analysis and uncertainty quantification. Such skills are at the foundation of the statistical profession and therefore should be represented and executed by statisticians [3]. Moreover, the technicalities of a chosen statistical approach must be accompanied by the ability to clearly communicate complex notions and ideas to decision makers and, equally important, to the public. Thus, statistical thinking should be an important component in all floors of the decision-making process (see Fig. 1).

First and foremost, statistical thinking has a role in the top floor where strategic plans are made. We would like to stress that statisticians should be involved in designing the strategic view of quality control, data collection and data management. The implementation of a specific strategy is at the tactical tier, and there too statisticians should be active as written in the sequel. Indeed, well explained statistical thinking can guide strategic discussions and point out promising directions, for example, recommending more efficient designs for data collection to support data-driven decision-making. At an early stage, statisticians should be involved in efforts to ensure quality control of data collection and data management. Tools from statistical process control can play a valuable role in such efforts. This is a crucial stage where an appropriate implementation according to best practices of data science will affect all future analyses. Clearly, statisticians can assume a leading role at the tactical and operational floors, where data collection and analyses are executed, and insights are generated. It is a stage where statisticians should be involved in analyzing the data. Insights need to be clearly and accurately presented to decision makers at the top floor.

In the industrial context, Deming [4] recommended placing statisticians in top level positions; “There will be in each division a statistician whose job is to find problems in that division, and work on them. He has the right and obligation to ask questions about any activity of the division, and he is entitled to responsible answers”. On top of the pyramid in Fig. 1, Deming envisaged the role of director of statistical methods [4]. Hahn, in his 2003 Youden address, generalized his experience at General Electric and described the role of the embedded statistician [5]. Building on these roles, Kenett and Redman [6] present an updated version of the integral role of statistics in the context of data science in organizations.

The structure of the rest of the paper is as follows. We begin by presenting the objectives of the roundtable. Subsequent sections describe the evolution of statistical activities in the early stages of the pandemic, discuss methods and tools for statistical intervention and describe the role of a statistician in the context of a pandemic. The next section details how the role of statisticians transformed as the pandemic evolved. Our Conclusions section summarizes the main points raised in the paper, followed by several recommendations.

## Main text

### Statistical analysis of coronavirus data: a roundtable

Acknowledging the potential benefit of sound statistical thinking in the decision-making process, a roundtable was organized by three past presidents of the Israel Statistical Association. Twenty colleagues from statistics and related areas participated in the event, which was hosted at the Technion by the Samuel Neaman Institute on 13 April 2021. The meeting was designed to provide a forum for discussion and exchange of ideas on the role that the profession should play in promoting evidence-based public policy and the way to deliver the information to decision makers. As a stimulus to discussion, the participants were invited to address questions such as:

- What are the main challenges in data collection flow and in leveraging statistical expertise in the COVID-19 Pandemic?
- What are the expected benefits of using statistical analysis of COVID data?
- What are the main barriers in developing and using statistical analytics in the pandemic?
- How can we overcome these barriers?
- What provisions should be taken to facilitate access to relevant data?
- What is the proposed role of academia in general, and statistical expertise in particular, in developing data analytics knowledge and capabilities?
- What should be considered success stories in using statistical analytics of Corona related data to guide policy decisions?

A report (in Hebrew) summarizing the round table is available at https://www.neaman.org.il/EN/Statistical-analysis-of-Corona-data-A-Roundtable.

The topics raised in the roundtable can be grouped into the following three categories:

1. The evolution of statistical activity as the pandemic unfolded.
2. The methods and tools for statistical intervention in decision-making—how to take the results of a statistical analysis and apply them to affect policy.
3. The role of statisticians and of statistics as a profession in a pandemic and more generally, how the discipline can aid in improving public policy.

This paper builds on the roundtable discussions and comments, providing several recommendations of general relevance to crisis management and public policy. The paper is organized according to the three categories mentioned above and aims to highlight the best way to leverage statisticians and statistics within the complex reality of emergency decision making. We take a holistic point of view, acknowledging that for statistics to have a significant impact, it must be actively represented in each of the decision-making domains.

### The evolution of statistical activity as the pandemic unfolded

In March 2020, the COVID-19 pandemic rapidly escalated from a minor news item about a virus outbreak in China to a full-blown national emergency. The Israeli government instituted a strict lockdown in an attempt to reduce spread of the virus. At this stage, the Israeli medical system needed diagnostic tools, hospitalization procedures and treatment protocols, as the magnitude of the pandemic and its requirements from the medical system were not well understood [7]. Policy strategies evolved in the light of population behavior and scientific knowledge as the pandemic evolved, leading to two more lockdowns. The vaccination campaigns, which began on the 19th of December 2020, led to a dramatic drop in the SARS-CoV-2 infection rate in Israel. The rapid curtailing of the pandemic was reflected in a gradual return to pre-COVID-19 activity; the roundtable, held in April 2021, was among the first of similar events to be held in person. Beginning in late June 2021, there was a resurgence of the pandemic, related to the incursion of the Delta variant and to the waning protection of the vaccine, which led to the decision in August 2021 to administer a booster dose.

The dynamic nature of the pandemic brought with it a changing landscape of challenges; the role of statisticians and biostatisticians evolved accordingly. In the hope of mitigating the dramatic impact of COVID-19 on society and the health and economic systems, it was important to address questions such as: How many individuals are infected with SARS-Cov2 virus? How many new infections are likely to occur in the week(s) ahead? What was the economic impact of a lockdown vs. no-lockdown decision? What was the effect of a lockdown on mortality from COVID-19? Do we need to implement new triage procedures that will be effective in preventing transmission of SARS-CoV-2 to patients and healthcare workers [8]? Health, social and economic data accompanied by appropriate analyses were crucial.

During the first phase of the pandemic, statisticians were involved in developing infrastructure, including data collection procedures and analysis approaches. There were some instances of direct involvement of statisticians in decision-making. Often, though, analyses by other scientists, sometimes with limited training and experience in statistics, were the basis for decisions. Examples of such constructive links between the statistical community and policy makers were provided by two important resources for the Ministry of Health: the statistical unit at the Gertner Institute for Epidemiology and Health Policy Research and the National Institute for Health Policy Research (NIHP).

These institutes and divisions are directed by statisticians or statistically savvy epidemiologists and include statisticians on their professional staff. Early in the pandemic, around March 2020, the Gertner Institute formed and coordinated a group of volunteers that included statisticians, data scientists and mathematicians. The group modeled the unfolding of the pandemic within the population using various mathematical and statistical tools and provided the Ministry of Health with estimates of the daily effective reproduction number. The group members focused daily on a variety of aspects of the pandemic such as: informing triage procedures and implementation using survival analysis; assessing treatment efficacy and social interventions using causal analysis; making fast, real-time decisions, such as recommending the number of ventilation machines required, combining applied probability and operations research approaches; supporting daily activities such as monitoring models and forecasts, and results of interventions by building informative, dynamic, and interactive dashboards; providing uncertainty assessment for all resulting numbers and recommendations.

Statistical input was important in guiding data collection. One example relates to the data from the labs carrying out PCR testing. Statisticians from the Gertner Institute stressed the need to report the age of tested subjects, which proved extremely useful for assessing the nature of the disease. A second concerns the data needed to support triage in ICU’s. Statisticians working with this problem highlighted the need for data on sojourn times, i.e. the length of time spent in different processing and disease stages, which are essential for modeling and prediction.

Much of the necessary data was collected from the health care providers in Israel, with supervision from their epidemiological research divisions. One of the efforts was to design and build a repository for storing COVID-19 data. Many challenges arose, such as the low reliability of diagnostic tools and the lack of unique criteria for defining variables (even the definition of “SARS-CoV-2 positive” was not unique and comparable). Indeed, there are numerous organizational units that collect and store data and are responsible for making them accessible. These groups include HMOs and hospitals, which use different data entry and storage platforms. In Israel, the collection and fusion of data from different sources is unique and has great potential. Efforts in this area are facilitated by the Israeli Ministry of Health. However, better standards would further improve data quality and rapid availability. Data often have inconsistent formats, making fusion difficult.

The NIHP acted quickly and in April 2020 issued a call for ‘COVID-19-specific’ research projects. After a rapid yet efficient review process about 20 projects were funded, the majority of which are based on collaboration with statisticians and/or epidemiologists. The NIHP also held a series of meetings (digital and in real life) where policy makers, leaders of the health services and researchers, including statisticians and data scientists, discussed various aspects of the pandemic such as challenges associated with obtaining suitable data for decision making and research and the characteristics of the dominant models used for predicting the development of COVID-19 in Israel. The head of the NIHP served as the statistician in a multidisciplinary academic group working on children and coronavirus which had substantial policy inputs [9].

The Israel Central Bureau of Statistics (CBS), together with the Ministry of Health (MoH) and the Gertner Institute, designed serological surveys in the heavily infected city of Bnei Braq to assess the extent and patterns of the infection. The CBS, MoH and the Gertner Institute applied scientific sampling methods to obtain representative samples, and also analysis methods that adjusted for the biasing effects of nonresponse, which are unavoidable in such surveys. The CBS was also at the forefront in providing timely data on the economic and social impact of COVID-19, launching four dedicated surveys of households, and 11 surveys of businesses in Israel. Timelines were accelerated to guarantee rapid availability of data to other government offices. Several standard reports were issued at double the usual frequency (for example, bi-weekly rather than monthly reports from the Labor Force survey) to enhance timeliness. Difficulty in obtaining data required application of more sophisticated statistical methods, including imputation and weighting, and the use of data from other sources (e.g., the income tax authority and credit card companies).

Statisticians were also involved in analyzing epidemiological data, clinical data, and data from basic research [10]. One study was based on a representative statistical sample with planned use of statistical principles, aimed to follow longitudinally the evolution of immunity related factors and to identify individual predisposing factors. The study design required defining surveillance procedures for frail groups of infected people and developing vaccination strategies. Israel was the world leader in the deployment of vaccinations [11], using the BNT162b2 vaccine developed by BioNTech in cooperation with Pfizer, and statisticians participated in, and initiated studies assessing vaccination effectiveness [12–14].

Statisticians contributed to efforts to find a modelling approach that would support extrapolation of information on epidemiological parameters such as mortality, fatality rates, incidence rates and predictors of disease evolution [15, 16]. The search for appropriate statistical approaches and tools in dealing with highly correlated covariates and variables has been a primary goal to help biomedical research identify COVID-19 risk factors.

### Methods and tools for statistical intervention

The data science approach is designed to support data-driven decision making. For decision making, whether in government, industry or academia, a data-driven approach should be taken in a context and therefore starts with a well-defined subject matter in mind. Appropriate and informative data must be collected or relevant data sources identified [17]. Several directions are possible for subsequent analysis. One option is to execute exploratory data analysis to better understand the data and to identify interesting trends or relationships. Another option is focused analysis that is directed toward answering a specific question. In both cases a variety of tools may prove useful, including statistical models and inference, machine learning, deep learning and uncertainty quantification.

**Fig. 2:**
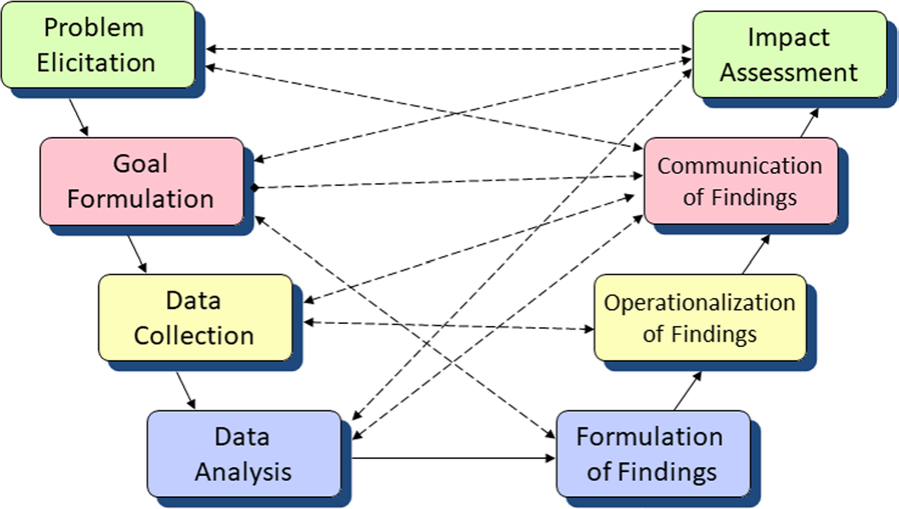
A life cycle view of statistics and data science. The outer loop of arrows indicates the process flow from Problem Elicitation to Impact Assessment and the influence and feedback loops that accompany it. The Impact Assessment often leads to highlighting additional problems, hence the arrow back to Problem Elicitation

The data science approach is represented by a life cycle perspective [18], see Fig. 2.

Figure 2, which describes the statistics and data science life cycle, includes a crucial data collection and management step. Indeed, the establishment of a "ground truth" database is essential, and it is important to collect various data sets that will give as broad a picture as possible. Furthermore, storing the data in an intelligent manner that allows easy access and querying by data scientists, domain experts and decision makers is of high importance. If possible, and found necessary, the different data sets should be fused into a single or several integrated data sets, which enhances the data analysis. We should enable data access and analysis using reliable and robust, but still flexible and efficient extract-transform-load procedures. In turn, appropriate data management allows the implementation of scientifically sound and statistically robust methods.

For example, estimating the reproduction number (*R*_*0*_) was crucial from the beginning of the pandemic, while understanding the effect of vaccines and planning successful vaccination strategies accordingly was relevant later on. Answering such scientific questions requires reliable data collection, prompt data availability and sound statistical analysis. Statisticians interested in providing insight through data analysis often found that the necessary data were not accessible, even in the context of protected data rooms.

Examples of reports which are based on data collection from different sources and the use of advanced statistical analyses mentioned above include [12, 16, 19, 20].

### The role of statisticians and of statistics as a profession in influencing public policy

The COVID-19 pandemic provides statisticians and policy-makers an opportunity to review the role of statistics within a wide range of policy domains. An emergency situation, whether a pandemic, an earthquake or an environmental disaster, requires rapid response from those in power. Often, as was the case with COVID-19, new challenges emerge as the emergency evolves. We are convinced that statisticians are highly qualified to address such challenges, owing to our training in analyzing and drawing sound conclusions from noisy data. Despite that expertise, the statistical community in Israel was often not involved in policy decisions. We can only conjecture on why that happened.

One major reason appears to be the lack of established points of contact, prior to the pandemic, between the community and those making decisions and determining policy. Faced with an emergency, it is expected that leaders turn to experts who are known and trusted. Such trust needs to be built from collaborative work to solve problems. Expertise alone is not enough; one needs to stand on a known track record of partnership. Although many statisticians were anxious to contribute to efforts to cope with COVID-19, most of them found they had almost no ability to influence policy. At the same time, leading scientists in disciplines not directly related to statistics and epidemiology were approached to contribute and influence policy decisions. What seems clear is that the efforts in Israel lacked sufficient multidisciplinary breadth, something needed in an event with health, social and economic impact.

The Central Bureau of Statistics (CBS), with an excellent track record in official statistics, was involved in questions of survey design. The serological survey in Bnei Braq, with many interesting and useful outcomes, was a noteworthy example. However, plans by the CBS to carry out national surveys to assess the spread of the pandemic, jointly with the Ministry of Health, never came to fruition. The CBS was also not involved in many other issues where its experts could have contributed.

Communication, both with decision makers and with the public, may have played a role. Statisticians excel in describing uncertainty, but that is often difficult to fully appreciate, especially by those who want a single sharp answer. The “limelight” factor may also have played a role, with members of the statistics community largely shying away from intense public exposure. Other professions also complained that their voice should have received more attention, but often with spokespersons who were very active in the media (e.g. the organization of public health physicians).

Statisticians often emphasize the need for thorough analysis, carefully examining all assumptions. There are many advantages to this approach. However, it may have made conventional statistical work too slow to meet the need for rapid analysis and response in emergency situations like a pandemic. That is unfortunate, as sharp statistical thinking and clear guidance are important in crisis times that require decisions in the face of huge uncertainty. Trust in data and its interpretation is a critical element in providing a clear and logical rationale underlying decisions in an emergency event like COVID-19. Without trust, compliance with instructions to the public is low, affecting the success of any policy.

On a deeper scientific level, the assessment of evidence should be considered a key competency of statistical work [21]. In a more general context, the growing role of data science needs to be considered [6], perhaps by getting statisticians to focus on a wider picture of information quality [17].

The situation in Israel during the first year of the pandemic was not altogether different from that in other countries. In Italy, statisticians were on the front lines of communication and became regular members of television news panels reporting on COVID-19. At the same time, the lack of data was lamented, and 1400 statisticians and researchers signed a petition to make COVID-19 data accessible. A special meeting of the Italian statistical society was held to deal with COVID-19 issues. Fricker and Rigdon [22] and Fricker [23] described aspects of the data collection and analysis efforts in the United States, noting that the effective investigation of disease outbreak is a domain where methods and tools can be improved. Fisher and Trewin [24] summarized statistical work in Australia, praising efforts of the Australian Bureau of Statistics, but also highlighting areas where improvements are needed, especially regarding the rapid availability of high-quality data. Among the consequences noted was a clear over-estimate, early in the pandemic, of the impact it would have on the health system. In the UK, the Royal Statistical Society formulated 10 recommendations for improvement, beginning with the need to present evidence and to be clear and open about data [25]. The final point stressed the need to apply careful post hoc evaluation of policies that were implemented, to determine if they had been successful.

In a recent and closely related paper, Ellenberg and Morris [26] provide an excellent discussion of the numerous challenges faced by statisticians, working alongside scientists from other disciplines, when aiming to understand the dynamics of COVID-19. They point out the critical role of statistical thinking in responding to the pandemic, including modeling the outbreak, tracking and reporting trends, characterizing the natural history of the disease, and evaluating interventions. They build on similarities and differences between the current pandemic and that of HIV/AIDS almost 40 years ago. The paper gained substantial attention and comments from leaders in biostatistics [27–29], arguing that statisticians have a unique opportunity to contribute during public health crises, to ensure that analyses are rigorous and based on proper data sources, inference is sound, and policy decisions are driven by the data. Important issues mentioned include the responsibility of statisticians in advocating for data quality and accessibility, the need to train statisticians in effective scientific communication with the media and the public, the importance of statisticians as scientific leaders and communicators, the role and voice of statistical professional bodies and the need to widen the interdisciplinary network of modelers [27–29]. The article and discussion further emphasize that the experience of the statistical community in Israel has many parallels abroad.

The lessons above pose challenges and call for action. We urge the statistical community in Israel and the nation’s policy makers to face them head on by taking steps to leverage the unique skills and experience of statisticians to influence policy in the future. In particular, there is a need for statisticians to be actively involved in all floors of the decision-making pyramid, starting with its most important top strategic floor (Fig. 1). Further, statisticians should be leaders in efforts to unify and standardize data collection, labeling and pipelines, considering aspects of security and privacy.

Three lessons from the COVID-19 experience seem particularly cogent. (1) The statistical community in Israel was not part of the inner circles of advice and influence during much of the pandemic. (2) To influence leaders in an emergency, experts need to build confidence and establish working relationships during periods of normalcy. (3) Statisticians should adopt a “push” strategy rather than waiting to be asked for help.

### Statistics in wave 4

The fourth wave of COVID-19 infection in Israel began at the end of June 2021. During that wave, a dramatic change occurred in the role filled by statisticians in support of evidence-based decision-making by the Israeli Ministry of Health. The group of statisticians, data scientists and mathematicians coordinated by the Gertner Institute, continued to analyze data and at this stage focused on the Israeli vaccination campaign. The fact that this work was done in collaboration with high official members of the Ministry lent it major importance in setting policy during Wave 4.

The first issue was to compare the protection of individuals who recovered from COVID-19 to others, both unvaccinated and vaccinated. The statistical analysis revealed that recovered individuals are protected in a similar fashion to individuals recently vaccinated with two doses [30]. An updated analysis found that recovered individuals also have waning immunity and has been the basis for requiring a booster for them, as well, after 6 months [31]. A second issue that required professional statistical analysis was to determine the level of vaccine protection against the Beta variant of SARS-CoV-2. The analysis demonstrated that, despite the concerns, the vaccine provides good immunity against the Beta variant. The data and analysis suggest that from 14 days after receiving the second dose, the efficacy is at most marginally affected [32].

The most influential project was to identify the waning immunity of the BNT162b2 vaccine during the fourth wave. Careful comparison of the rate of infection as a function of vaccination time demonstrated that immunity against the Delta variant, which dominated the fourth wave, wanes in all age groups 6 months after the second vaccine dose [33]. The statistical analysis was instrumental in assisting the Ministry to administer a third dose to individuals who received their second dose more than 5 months ago. Israel was the first nation in the world to adopt this booster policy, which enabled control of the outbreak without a lockdown. In addition, the group used the real life data from those who received the third dose to estimate the protection it provides [34]. The outcomes of the last two studies have enabled other nations to make similar decisions regarding a booster dose. The analyses were presented by the Ministry of Health to the US Federal Drug Administration and were key pieces of evidence in their decision to advocate a booster dose for older individuals and for those in high-risk jobs [35].

Partnering with the CBS has also accelerated and expanded to conditions beyond COVID-19. The Ministry of Health hopes to run, with the methodological support of the CBS, a national non-probability survey to estimate the prevalence of viral disease in the Israeli population.

## Conclusions

Given complex situations such as the one we observed during the COVID-19 pandemic, data-driven policies are essential. This requires access to data that is actionable. In addition, data-based policy recommendations that can support decision makers such as government ministers, hospital managers, education system administrators and economic system leaders require the use of validated models that can be used for analyzing the evolution of the pandemic, the effects of interventions and for predictions.

The challenge of reaching well informed decision-making stretches far beyond having the “right”, high quality data or good models. A successful data-driven approach to decision-making requires efficient multidisciplinary collaboration which in turn, depends crucially on having trust and common language between disparate domains. This is true not just for supporting the Ministry of Health, but also for other government branches such as the Ministry of Finance, the Ministry of Economy and Industry, the Ministry of Labor, Social Affairs, and Social Services, and the Ministry of Justice. All seek or should seek data-driven decision making and all are relevant in crisis times as the one we experienced recently. This is possible only when there are already information systems in place for collecting, storing and presenting data. It requires pipelines for connecting data from diverse sources for joint analysis. Data collected from different sources need to be structured and definitions must be consistent and agreed upon, and relate to the same or at least similar time periods. Building a common language takes time and should start immediately.

Thus, good channels of communication are essential for reliable data and insightful analyses to reach the right eyes and ears and have an essential influence on action. Moreover, adopting a data science life cycle point of view has great potential in establishing efficient multidisciplinary working procedures that naturally lead to well informed decision-making. Therefore, practicing daily collaboration of multidisciplinary teams in “normal” times will have a huge benefit in crisis time, resulting in seamless execution of data-driven decision-making thanks to the trust and common language built along the way. The academic statistical community, the Central Bureau of Statistics, the Gertner Institute and the National Institute for Health Policy Research are already geared to such multidisciplinary working procedures, but other ministry and public organizations need to join in.

## Recommendations

1. Lobby for active participation of statisticians in decision-making forums.
2. Establish a pool of statisticians from academia and industry, as well as of other experts, that will work routinely in normal times in multidisciplinary teams together with policy decision makers. This requires the establishment of a policy committee that can serve as a contact point between decision makers who seek evidence-based policy and statisticians who are interested and ready to share their knowledge and expertise.
3. Improve the organization and allocation of statistical tasks in the face of a health emergency. This could also be handled by the policy committee.
4. Government ministries and other public agencies can fund scholarships for students to further support such an initiative, for example within the framework of the successful Science and Policy Fellowship Program Mimshak [36].
5. Establish national standards for health records, including treatment of free text entries.
6. Educate stakeholders to appreciate the importance of standards and statistical thinking and analysis.
7. Hold professional meetings to discuss challenges and debate possible solutions.
8. Enhance presence in the media, where statistical expertise must be brought to the public in language that is understandable to a broad audience. “Push” rather than wait to be called.
9. Discuss the *Nature* “model manifesto” [37] and the role of academic research in a variety of academic and non-academic circles [38].
10. Train the next generation of statisticians and data scientists to have the needed skills (statistical and communications) to aid in decision-making during crisis.

These recommendations are presented with a forward-looking perspective. The role of statisticians is crucial in the evaluation of the impact of COVID-19 on the population and in preventing a resurgence of new COVID-19 waves. Many statistical issues are critical in the analysis of public health data; consequently, statisticians should have access to such data. Healthcare systems, in general, should be prepared to absorb a sudden deterioration of patients in areas such as chronic pathologies, which could result from cure avoidance or postponement of screening examinations. For example, careful evaluation and comparison of historical data with actual data, based on statistical predictive models is needed. Statisticians should be essential partners in implementing data-driven public health due to their knowledge of sampling strategies; this is an essential starting point in building statistical expertise and is needed in designing surveillance systems, which depend heavily on sampling strategies to provide early alert to the onset of infectious diseases and to map indices of disease spread [39, 40].

In conclusion, effective use of statistics emerges as crucial in times of crisis and in particular, for evaluating the impact of COVID-19 on the population and limiting the impact of new COVID-19 waves. We fully agree with Ellenberg and Morris [26] and their discussants that for many of the unusual challenges posed by COVID-19, sound statistical work is instrumental to find good solutions. Consequently, statisticians should be important contributors to collecting, analyzing and interpreting data related to the pandemic and in translating that work into sound policy and decisions. The daily contributions of statisticians described above, started early in the pandemic and continuing today, evolved to have significant impact during wave 4 of the pandemic. Such impact was possible due to trust and common language built from the daily communication with decision makers. This is an excellent illustration of the dramatic and beneficial impact that informed statistical analysis and advice can have on public policy in a time of crisis.

## Data Availability

Not relevant for this paper.

## Abbreviations

AIDS: Acquired Immune Deficiency Syndrome
CBS: Central Bureau of Statistics
HIV: Human Immunodeficiency Virus
HMO: Health Maintenance Organization
ICU: Intensive Care Unit
MoH: Ministry of Health
NIHP: National Institute for Health Policy
PCR: Polymerase Chain Reaction
SARS: Severe Acute Respiratory Syndrome
UK: United Kingdom
